# Case Report: Decrypting an interchromosomal insertion associated with Marfan’s syndrome: how optical genome mapping emphasizes the morbid burden of copy-neutral variants

**DOI:** 10.3389/fgene.2023.1244983

**Published:** 2023-09-21

**Authors:** Maria Clara Bonaglia, Eliana Salvo, Manuela Sironi, Sara Bertuzzo, Edoardo Errichiello, Teresa Mattina, Orsetta Zuffardi

**Affiliations:** ^1^ Cytogenetics Laboratory, Scientific Institute, IRCCS E. Medea, Lecco, Italy; ^2^ Bioinformatics, Scientific Institute, IRCCS E. Medea, Lecco, Italy; ^3^ Department of Molecular Medicine, University of Pavia, Pavia, Italy; ^4^ Neurogenetics Research Center, IRCCS Mondino Foundation, Pavia, Italy; ^5^ Medical Genetics Unit, University of Catania, Catania, Italy; ^6^ Clinic G.B. Morgagni, Catania, Italy

**Keywords:** *FBN1*, *ROBO2*, chromothripsis, complex chromosome rearrangement, intellectual disability

## Abstract

Optical genome mapping (OGM), which allows analysis of ultra-high molecular weight (UHMW) DNA molecules, represents a response to the restriction created by short-read next-generation-sequencing, even in cases where the causative variant is a neutral copy-number-variant insensitive to quantitative investigations. This study aimed to provide a molecular diagnosis to a boy with Marfan syndrome (MFS) and intellectual disability (ID) carrying a *de novo* translocation involving chromosomes 3, 4, and 13 and a 1.7 Mb deletion at the breakpoint of chromosome 3. No *FBN1* alteration explaining his Marfan phenotype was highlighted. UHMW gDNA was isolated from both the patient and his parents and processed using OGM. Genome assembly was followed by variant calling and annotation. Multiple strategies confirmed the results. The 3p deletion, which disrupted *ROBO2*, (MIM*602431) included three copy-neutral insertions. Two came from chromosome 13; the third contained 15q21.1, including the *FBN1* from intron-45 onwards, thus explaining the MFS phenotype. We could not attribute the ID to a specific gene variant nor to the reshuffling of topologically associating domains (TADs). Our patient did not have vesicular reflux-2, as reported by missense alterations of *ROBO2* (VUR2, MIM#610878), implying that reduced expression of all or some isoforms has a different effect than some of the point mutations. Indeed, the *ROBO2* expression pattern and its role as an axon-guide suggests that its partial deletion is responsible for the patient’s neurological phenotype. Conclusion: OGM testing 1) highlights copy-neutral variants that could remain invisible if no loss of heterozygosity is observed and 2) is mandatory before other molecular studies in the presence of any chromosomal rearrangement for an accurate genotype-phenotype relationship.

## Introduction

Marfan syndrome (MFS, MIM # 154700) is a multisystem autosomal dominant connective tissue disorder caused by heterozygous variants of the fibrillin-1 gene (*FBN1,* 15q21.1). Both dominant-negative effects and haploinsufficiency are reported in its pathogenesis (Aubart et al., 2018) and are expected by the constraint metrics of *FBN1* (Z = 5.06; pLI = 1; gnomAD v2.1.1).

Confirmation of *FBN1* alterations, mainly missense and loss of function (Lof) variants or rare chromosomal rearrangements (Colovati et al., 2012; Dordoni et al., 2017; Schnause et al., 2021), is achieved in approximately 90% of cases meeting the Ghent II nosology criteria applied for the clinical diagnosis of MFS (Loeys et al., 2010; Zeigler et al., 2021). Alterations of *FBN1* linked to MFS have also been identified outside the *FBN1* coding region and were proven to be causal through functional analysis (Guo et al., 2023). However, in approximately 10% of patients with distinctive clinical signs of MFS, including positivity to GHENT II criteria, no alteration of *FBN1* is detectable by routine DNA investigation. In this regard, the familial case reported by Pagnamenta (Pagnamenta et al., 2022) is exemplary: a 1.97 Mb inversion with a distal breakpoint in intron 4 of *FBN1* was highlighted after years of genetic testing in the mother and proband, who were recruited with a diagnosis of “familial thoracic aortic aneurysm disease.” We present a similar case of a male boy long suspected of suffering from MFS complicated by ID. A *de novo* complex rearrangement involving chromosomes 3, 4, and 13 was discovered at amniocentesis; however, the search for *FBN1* alteration explaining his postnatal MFS phenotype gave negative results after MLPA, array-CGH, and gene panel sequencing. The involvement of chromosome 15 with a copy-neutral insertion of a portion of *FBN1* at the breakpoint of chromosome 3 was eventually detected by OGM. This case not only confirms the superiority of this technological approach in revealing structural variants (SVs) but also stresses the burden of the copy-neutral structural variants underlying genetic disorders.

## Material and methods

### Clinical report

**FIGURE 1:**
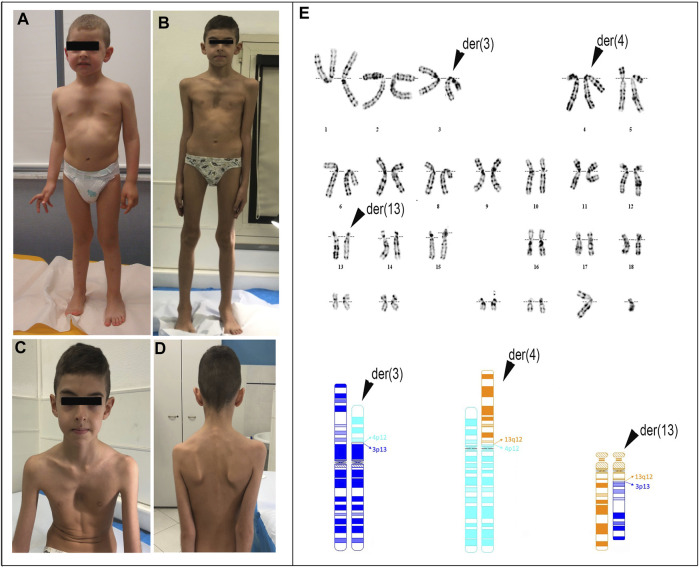
Photographs of the patient at the age of 3 years and 1 month **(A)** and 7 years and 9 months **(B–D)**. Note: pectus excavatum, arachnodactyly, winged scapula, facial dysmorphisms including dolichocephaly, and mildly asymmetric face (left < right) are indicated. Conventional cytogenetics analysis: **(E)** (upper) G-banding karyotype showing the three-way translocation 46,XY,t(3;13;4)(p13,q12,p12); (bottom) partial ideograms showing the normal and derivative (der) chromosomes (chr) 3 (dark blue), 4 (light blue), and 13 (orange) as well as the fragments participating in the rearrangement.

**FIGURE 2:**
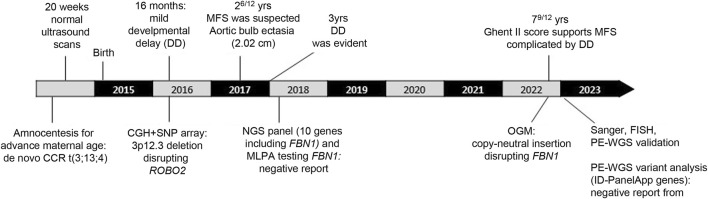
Timeline of clinically relevant patient data and related diagnosis.

The patient, a 7-year-old male (Figures 1A–D), is the fourth child of healthy non-consanguineous parents. The proband was born full-term via normal delivery, weighing 3,600 g (50th–75th centile) and was 49 cm long (25th centile), to a 41-year-old mother and a 43-year-old father. Amniocentesis showed a male karyotype with a *de novo* and apparently balanced rearrangement involving three chromosomes [46,XY,t(3;13;4)(p13;q12;p12)dn] (Figure 1E). Ultrasound at 20 weeks gave normal results. Developmental delay was evident since the age of 16 months; therefore, array-CGH investigation was requested to investigate any possible imbalance associated with the chromosome rearrangement. A deletion of 1.65 Mb at 3p12.3, apparently coinciding with one of the breakpoints of the rearrangement, was detected and confirmed by FISH analysis, although its genetic content failed to explain the delay in development. At 2^6/12^ years of age (Figure 1A), MFS was suspected because of a height >97th centile, arachnodactyly, scoliosis, bilateral joint hyperlaxity, marked pectus excavatum, mild mitral valve prolapse, and aortic bulb ectasia with a 2.02 cm diameter. Chest X-ray highlighted an enlarged left heart. At 3 years of age, global developmental delay was evident, mainly affecting cognitive and communication skills but no specific tests were performed. His weight was 14.8 kg (50th centile), height 102 cm (97th centile), OFC 49 cm (10th–25th centile), and arm span 102 cm. Thumb sign, pes planus, thoracolumbar kyphosis, and reduced elbow extension were observed. Gene panel sequencing, including *COL5A1*, *COL5A2*, *COL1A1*, *COL1A2*, *COL3A1 TNXB*, *TGFBR*, *TGFRB1*, *TGFBR2*, and *FBN1*, did not identify pathogenic variants. MLPA showed no exonic deletions or duplications at *FBN1*. At the age of 7^9/12^ years (Figures 1B–D), his height was 139.5 cm (>>97th centile), arm span 142 cm, weight 22 kg (10th centile), and OFC 50 cm. For positivity to GHENT II criteria, the patient’s score was 8 (Supplementary Table S1), which supported the clinical diagnosis of MFS complicated by developmental delay. Renal ultrasound to investigate whether haploinsufficiency of *ROBO2* was associated with VUR2 did not reveal any abnormality. A timeline of clinically relevant patient data and related diagnosis is shown in Figure 2.

### Cytogenetics and microarray investigations in the trio

Karyotyping was performed at a resolution of ∼550 bands. Array-CGH was performed using the CGH + SNP microarray (180k, Agilent). All nucleotide positions refer to the human genome, assembly (hg38). Data analysis was performed using Agilent Cytogenomics V.5.2.0.20.

### Optical genome mapping (OGM) in the trio

UHMW gDNA was isolated from 1.5 million cultured lymphoblastoid cells from the patient and his parents using an SP Cryopreserved Cell isolation kit (Bionano Genomics, San Diego, California, United States) according to the manufacturer’s instructions. gDNA was labeled with a direct label (DL) and Stain DNA Labeling Kit using Direct Label Enzyme 1 (DLE-1) and DL-green fluorophores, loaded on a nanochannel chip, and analyzed on a Saphyr instrument (Bionano Genomics). A minimum of 320 Gb of data were acquired. *De novo* genome map assembly was performed using Bionano Solve software V.3.7. SVs (based on the assembled genome maps) and CNVs (based on molecular coverage) were called against the human reference genome (GRCh38/hg38). Analysis of these data was performed with Bionano Acces V 1.7.0 s and Bionano tools on the Saphyr Compute On Demand server. The following filtering confidence thresholds were applied: insertion/deletion; 0, inversion; 0.7, duplications; −1, intratranslocation; −1 and 0.05, intertranslocation; −1 and 0.05, and CNV; 0.99. A masking filter was applied. For CNV_calls, only segments >500 kb were considered. SVs_calls were filtered using Bionano’s human control sample SV database containing variants collected from > 300 human genomes with no reported disease phenotypes. Only SVs below 1% were taken into consideration.

### Confirmation of OGM analysis by pair-end whole-genome (PE-WGS), sanger sequencing, and FISH

Genomic DNA from the proband’s and parents’ blood was sequenced using an Illumina Hiseq 2000 platform, employing a 30× PCR-free paired-end WGS protocol. Reads were mapped to the human reference genome GRCh38/hg38 using BWA (Li and Durbin, 2009). SVs were called using Lumpy (Layer et al., 2014) and Delly (Rausch et al., 2012) and were visualized and manually checked in the Integrative Genomics Viewer (IGV) genome browser to identify sample-specific SVs. Variant calling was obtained using the recommended best practices, in agreement with the Genomic Analysis Tool Kit v3.7-0 (GATK). Segment junctions were confirmed by PCR and Sanger sequencing using primers listed in Supplementary Table S2. FISH analysis was performed using the locus-specific probe RP11-552E10 (Empire Genomics).

### Fusion gene prediction

To determine fusion-genes candidates, we used ExPASy’s Translation Tool (http://web.expasy.org/translate/), and the generation of novel fusion protein motifs was predicted using ScanProsite (http://prosite.expasy.org/scanprosite/).

### Topologically associating domains (TADs) analysis

The search for TADs was performed using a web-based 3D Genome Browser (Yardımcı and Noble, 2017).

## Results

### Conventional cytogenetics analysis

The patient harbored three derivative chromosomes, [46,XY,t(3; 13; 4) (p13,q12,p12)dn, Figure 1E], and a 3p12.3 interstitial deletion of approximately 1.7 Mb affecting the paternal chromosome: arr[GRCh38]3p12.3(75,839,392_77,548,980)x1dn (Supplementary Figure S1; Supplementary Table S3). This latter removes exons 1 to 8 (RefSeq NM_001395656.1) of *ROBO2.*

### Deciphering complex chromosomal rearrangement using OGM

**FIGURE 3:**
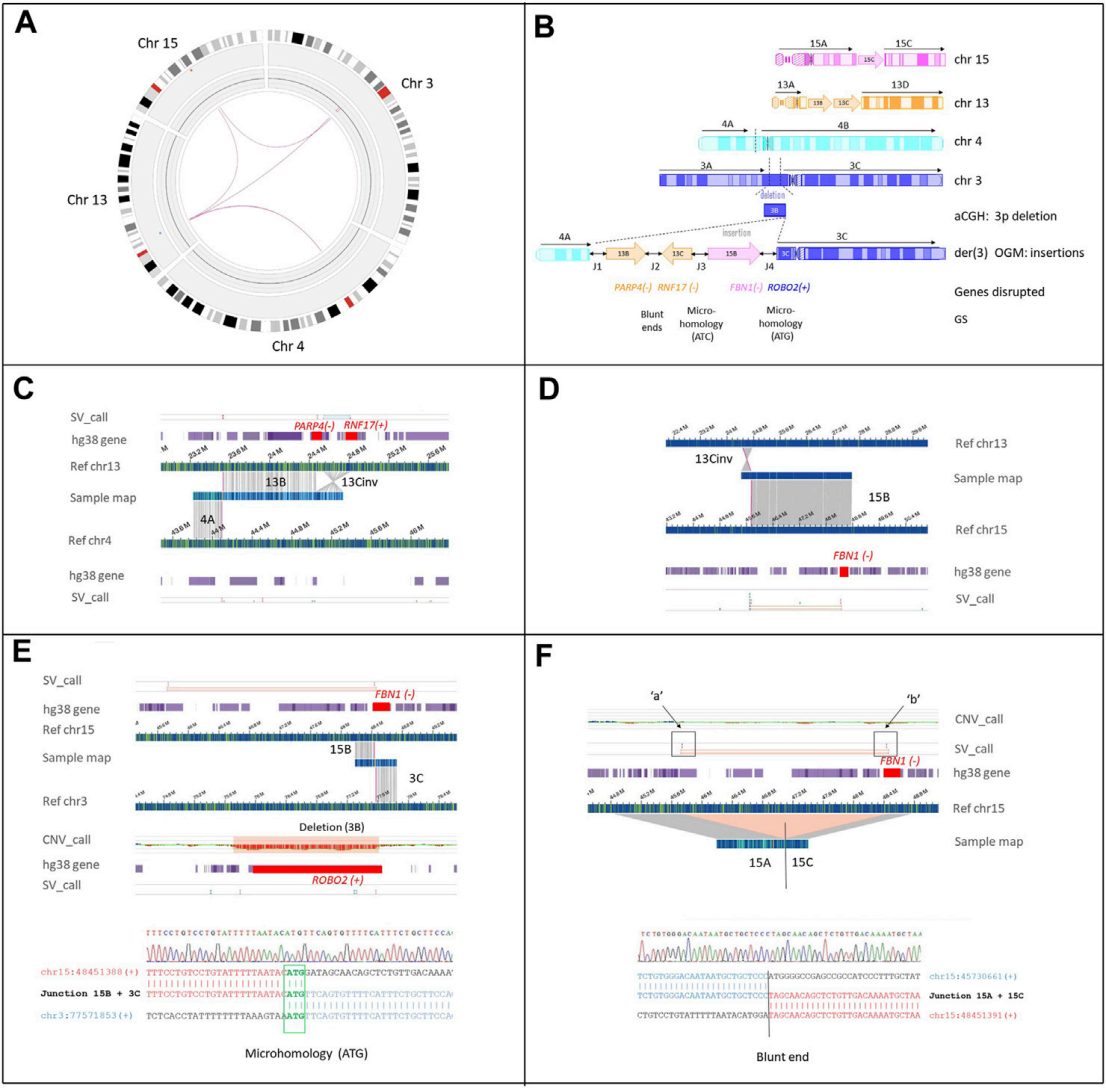
Cryptic interchromosomal insertions as detected by OGM SV calling. **(A)** OGM circos plot of chromosomes 3, 4, 13, and 15 involved in the CCRs. Interchromosomal insertions from chromosomes 13 and 15 to chromosome 3, [t(3;13), t(3;15)] are indicated by magenta lines. **(B)** Schematic representation of the rearrangement. The ideograms of the four chromosomes involved in the rearrangement show both the inserted (depicted as arrows: 13B and 13C, inversely aligned to reference chromosome 13: 13Cinv) and translocated fragment (4A) transposed into the der(3). The truncated protein-coding genes and their breakpoint genomic signatures (GS) are shown; + and − at the left of the gene names indicate their transcription orientation (hg38). The junctions (double-black arrows) between the transposed fragments are numbered from 1 to 4 (J1-4), as in Table 2. **(C)** Genome maps of the patient (sample map) aligns to two contiguous areas of reference: chromosome 13 corresponding to fragment 13B and 13C, the latter inversely aligned to reference chromosome 13 (13Cinv), and the area of reference chromosome 4 corresponding to fragment 4A. The breakpoint that joined segments 13B and 13Cinv disrupted *PARP4* and *RNF17* (red square in the hg38_gene track), leading to a putative fusion transcript PARP4(-)-RNF17(-) (see also Supplementary Figure S3) **(D)** Sample fusion map between 13Cinv and the region that aligns to chromosome 15 (segment 15B). The SV algorithm breakpoint (SV_call) indicates that the proximal breakpoint of segment 15B to which segment 3C is joined (see the following panel E), interrupts *FBN1* (highlighted in red square in the hg38_gene track) at intron 46, leading to transposition of the distal 20 exons to chromosome 3. **(E)** Copy number track showing the 3p deletion picked up by the copy number algorithm (CNV_call). The deletion disrupts the ROBO2 gene (highlighted by the red square). The SV_call demonstrates that fragment 15B, including the distal region of *FBN1*, has been inserted to der(3) where the 3p deletion has occurred. Bottom panel: breakpoint junction sequence (J3) confirmed by Sanger sequencing. Microhomology of 3bps (ATG) between 15B and 3C sequences is highlighted in green. **(F)** Genome map of the patient containing the 3’ portion of *FBN1* (ex1-45) on fragment 15C. The SV_call and the concurrent absence of CNV_call show that the 15B inter-label region (45,720,396 and 48,459,546, marked “a” and “b”, respectively) is not lost but rather incorporated into another chromosome, i.e., the der(3). The sample map shows the fusion of fragments 15A and 15C by blunt end repair. Further details are provided in Table 1 and Table 4.

OGM analysis confirmed the cytogenetic and CMA results, and also identified the involvement of a fourth chromosome, chromosome 15 (Figure 3A).

Chromosomes 3, 4, 13, and 15 were fragmented into 12 segments (Supplementary Table S4). Three of them, two derived from chromosome 13 (Figures 3B–D) and one from chromosome 15 (Figures 3B,E,F), were inserted with an apparently random order and orientation within the short arm of the derivative chromosome 3. FISH analysis with probe RP11-552E10 (Supplementary Figure S2) and breakpoint junctions mapping demonstrated that fragment 15B was inserted into the 3p12 deletion with the same orientation as the reference genome (Figures 3E,F). Moreover, more precise deletion breakpoints were obtained, showing the removal of exons 1–14 of *ROBO2* and not 1–8 as estimated by array-CGH (Supplementary Figure S1). The 3’ portion of *ROBO2* (NM_001395656.1), starting from exon 15, was joined to the 3’ portion of *FBN1*, namely, exons 46–66 of *FBN1* (MIM *134797, NM_000138.5). This rearrangement generated a putative fusion gene, which did not preserve the reading frame and, thus, was predicted to trigger nonsense-mediated mRNA decay (NMD) (Figure 3E; Table 1). Two other RefSeq coding genes [*PARP4*:MIM*607519 and *RNF17:*MIM *605793)] were interrupted by the insertion of chromosome 13 into chromosome 3p (Figure 3B). Unlike fragment 13B, fragment 13C was inserted with an inverted orientation and, when joined at fragment 13B, created a PARP4-RNF17 in-frame fusion transcript (Supplementary Figure S3).

**TABLE 1:** Breakpoint junctions verified by PE-WGS and Sanger sequencing (*SD: segmental duplication with 90%–98% similarity).

Derivative chromosome	Breakpoint junction	Fragments joined (orientation)	Genomic coordinates (hg38) of breakpoint junction (orientation)		Sequence signature at breakpoint junction	Repeats at breakpoint junction	Genes fusion at breakpoint junction (orientation)
der 3	J1	4A(+) +13B(+)	chr4:44104796(+)	chr13:23529127(+)	microhomology 5 bp (CCAGG)	LINE(L1)/LTR(ERV1)	
	J2	13B(+) + 13C(-)	chr13:24468640(+)	chr13:24812394(-)	blunt ends	SINE(Alu)/LINE(L1)	*PARP4*(-)/*RNF17*(-)
	J3	13C(-) + 15B(+)	chr13:24468642(-)	chr15:45730678(+)	microhomology 3 bp (ATC)	SINE(Alu)-SD*/--	
	J4	15B(+) + 3C(+)	chr15:48451388(+)	chr3:77571853(+)	microhomology 3 bp (ATG)		*FBN1*(-)/*ROBO2*(+)
der 4	J5	13D(-) + 4B(+)		chr4:44104800(+)		LINE(L1)	
der 13	J6	13A(+) + 3A(-)	chr13:23529137(+)			LTR(ERV1)	
der 15	J7	15A(+) +15C(+)	chr15:45730661(+)	chr15:48451391(+)	blunt ends		

### Breakpoint-junction analysis using PE-WGS and sanger sequencing

All the OGM breakpoint-junctions (Table 1) were confirmed by visual inspection of the locations of discordant paired-read and soft-clipped reads using IGV, and, whenever possible, refined by PCR and Sanger sequencing (Supplementary Figures S4–S7). Three junctions were characterized by microhomology of 3–5 bases and two by blunt ends (Table 1). The occurrence of four derivatives, the number of non-clustered breakpoints with only one cis-junction (between fragments 13B and 13C), the deletion present at one junction, and the breakpoint characteristics suggest that the CCR could fit a classification of chromoanagenesis, specifically chromoplexy.

### Single nucleotide variant (SNV) analysis using PE-WGS

WGS analysis did not identify pathogenic/likely pathogenic SNVs according to the ACGM guidelines (Richards et al., 2015) in known ID-associated genes (https://panelapp.genomicsengland.co.uk/panels/285/).

## Discussion

There is growing evidence that OGM can integrate all forms of SV, even throughout complex loci, thanks to the uninterrupted assembly of long-range molecules, allowing anchoring and resolving of most SVs regardless of sequence composition (Porubsky et al., 2022).

Indeed, in the case we studied, the insertional translocation of 2.7 Mb leading to the breakage of *FBN1* has been detected thanks to OGM and, after 7 years of vain investigations, explained the reasons for the main patient’s features, i.e., those coinciding with the Marfan phenotype. On the other hand, no clear basis for the patient’s ID could be highlighted. PE-WGS revealed no like-pathogenic or pathogenic SNVs in known ID-associated coding genes, thus pointing to the other genes altered by the rearrangement, namely, *ROBO2, PARP4*, and *RNF17*. *ROBO2,* which was partially lost as a consequence of the 3p deletion, and is of interest, being mainly expressed in the brain (GTEx, V6 release) and intolerant to Lof variants (pLI = 1; o/e = 0.08; gnomAD v2.1.1). Furthermore, a significant association of rs7642482, near *ROBO2*-3’, with expressive vocabulary in infancy was demonstrated (St Pourcain et al., 2014), whereas a decreased expression was observed in the brains of individuals with autism spectrum disorders (Suda et al., 2011). These findings are in agreement with the *ROBO2* function as a receptor for SLIT2, and probably SLIT1, which are thought to act as a molecular guide in cell migration, including axonal navigation at the ventral midline of the neural tube and axon projection in different regions during neuronal development (RefSeq NM_002942). To date, heterozygous *ROBO2* pathogenic variants have been associated with autosomal dominant VUR2. The ultrasound scan of the patient’s abdomen, specifically requested to highlight any possible alteration of the kidney and urinary tract, associated with the partial loss of *ROBO2*, did not reveal any abnormality, and no clinical signs such as recurrent urinary tract infections were evident.

SNVs associated with VUR2 are located along the entire gene (Supplementary Figure S8) and are mainly of the missense type, suggesting that VUR2 syndrome is the result of a dominant-negative effect. In our patient, the deletion involving *ROBO2* removes exons 1–14 (Figure 3E; Supplementary Figure S1), which encode most of the extracellular domains of the protein (Supplementary Figure S8). Analysis of the spared sequence revealed the presence of a start codon at position +18 of exon 15, possibly indicating that the C-terminal portion of the protein is translated. This portion retains the transmembrane domain and cytoplasmic region, which is characterized by three intrinsically disordered regions (Supplementary Figure S8). It is, however, difficult to speculate on the effective production and functionality of this truncated protein. Even if translated, the protein is predicted to lack the signal peptide, a short N-terminal sequence that drives trafficking to the cell membrane via the endoplasmic reticulum (Guna and Hegde, 2018). Mutations or deletions in the signal peptides of other human proteins were shown to result in reduced protein targeting to the cell membrane, retention in the endoplasmic reticulum, or very low-level intracellular expression (Albers et al., 2014; Uetz-von Allmen et al., 2018; Potorac et al., 2019). In any case, even if translated and spared from degradation, the protein would miss the extracellular domains required for interaction with SLIT2, an important mediator of neuronal migration (Bagri et al., 2002). Thus, in our patient, the deletion is likely to have resulted in a reduced amount of functional ROBO2 protein, which may have affected his neurological development by impairing the axon-guiding function, as demonstrated in the anterior cingulate cortex (Suda et al., 2011) and in lymphocytes of individuals with autism (Anitha et al., 2008).

*PARP4* and *RNF17*, which are also broken by the transposition from 13q to 3p, are not so far disease-associated, and both of them are almost not expressed in the brain. The good tolerance of *PARP4* to Lof variants (pLI = 0; gnomAD v2.1.1) makes its involvement in the intellectual disability of the patient unlikely. In contrast, *RNF17* appears highly intolerant to Lof variants (pLI = 1; o/e = 0.03; gnomAD v2.1.1); however, according to its expression limited to testis, may be involved in spermiogenesis only. In conclusion, an explanation for the ID of the patient is missing, although *ROBO2* appears to be the best candidate. Moreover, we must consider that the reshuffling of topologically associating domains (TADs) may have caused misexpression of intact genes around the breakpoints, especially those within 100 kb (Schöpflin et al., 2022). In silico analysis of the three-dimensional organization of chromatin (Supplementary Figure S9) showed that none of the identified rearrangement breakpoints altered TADs organization or occurred within highly conserved non-coding sequences associated with developmental regulators (Lowther et al., 2022). However, we cannot rule out that other genes in the 100 kb surrounding breakpoints have unbalanced allelic expression (Schöpflin et al., 2022).

### The rearrangement

The rearrangement we investigated was *de novo* and of complex type. It is classifiable as a chromoplexy, an event characterized by the exchange of large fragments between chromosomes with or without loss of material (Zepeda-Mendoza and Morton, 2019). Indeed, for years, three breakpoints, one in the recipient chromosome and two in the donor, have been considered as the basis for insertion occurrence (Madan, 2013). However, in a limited number of cases, NGS approaches have shown that insertions are events in which several pieces from localized regions of one or more donor chromosomes are mixed and inserted in a disordered arrangement within another recipient chromosome, or in two recipient chromosomes, or in another region of the same donor chromosome (Gu et al., 2016; Kato et al., 2017; Dong et al., 2021). A deletion at the inserting position has been reported in both *de novo* and inherited rearrangements, with the insertion being either copy-number neutral as in our case, or copy-number gain (cases 1 and 2 in Kato et al., 2017 and Cplex4, Cplex9, and Cplex12 in Gu et al., 2016). Breakpoints’ definition in some complex rearrangements, especially chromoplexis events, showed overlaps with different repeat classes (Schöpflin et al., 2022). In our case, five out of seven breakpoints fell within LINE, SINE, and LTR repeats, with a signature of microhomology in two cases, and blunt-end in one (Table 1). In particular, the inversion of segment 13C, with retrotransposons to the breakpoints, points to non-allelic homologous recombination as the preferential mechanism of formation of this type of rearrangement (Porubsky et al., 2022).

## Conclusion

Our patient has received a clinical diagnosis of MFS since he was 2^6/12^ years old. However, the lack of confirmation at the molecular level and the concomitant presence of ID created uncertainties about the real cause of the clinical condition. OGM, which was performed 5 years after the first molecular investigations ended the diagnostic odyssey by showing that the patient was actually suffering from MFS due to the transposition of part of the *FBN1* gene from 15q to 3p, even if the basis of the patient’s ID remains vague and considering possible alterations of the TADs as a consequence of the rearrangement’s breakpoints. While more evidence is needed, *ROBO2* appears to be the best candidate for the ID observed in our patient.

Our study further emphasizes the role of neutral SVs in missing diagnoses of Mendelian disorders, including those that are clinically and molecularly confident. This is the case for subchromosomal-size inversions for which OGM has been shown to be much more frequent than previously estimated (Porubsky et al., 2022). OGM highlights that even copy-neutral insertions increase the burden of genetic disorders, demonstrating that some of them are found in regions refractory to sequencing (Sabatella et al., 2021; Yang and Hao, 2022; Zhang et al., 2023). A separate condition concerns the so-called copy-neutral loss-of-heterozygosity (CN-LoH). This condition has been reported in aging, cancer, and increasingly in congenital disorders. In all cases, variants with a lower cell fitness, disease variants, or chromosomal imbalances can be removed by somatic recombination, resulting in segmental uniparental disomy and CN-LoH (Loh et al., 2020; Papenhausen et al., 2021). CN-LoHs are detected through trio SNP analysis and are unquestionable of postzygotic origin. However, the expected mosaicism with the cell line presumably present in the zygote or at early embryogenesis is not always detected, sometimes making it difficult to correlate the CN-LoH region with the patient’s phenotypic abnormalities. At least in the case of CN-LoHs in mosaic with SVs for the same chromosome regions, OGM technologies could indeed be superior to other sequencing techniques (Noyes et al., 2022).

This study provides further overwhelming evidence that chromosomal rearrangements, although not necessarily complex, require OGM testing, even before any other molecular investigation. This approach might be suitable both in the presence of congenital disorders and in apparently healthy infants, considering the long-term morbidity that can be unpredictable at birth (Li et al., 2014; Halgren et al., 2018; Fjorder et al., 2019).

Even if the breakpoint sequence is not provided, the OGM software we used can capture and highlight complex genomic rearrangements in a very intuitive and effective manner. The combination of the CNV and SV pipelines allows one to both highlight unbalanced variants >500 bp and to reconstruct the final order and orientation of the displaced regions. In contrast, short-reads genome sequencing, without knowing *a priori* which regions have to be investigated, requires endless manual visual inspection with the risk of losing regions masked by segmental duplications/high copy repeats that are not captured by the methodology. Based on the present study, the use of OGM before any other molecular analysis is recommended for the complex chromosomal rearrangement, and perhaps for the apparently simple ones, both associated with congenital clinical disorders or present in apparently healthy newborns, taking into consideration long-term morbidity that can be unpredictable at birth (Li et al., 2014; Halgren et al., 2018; Fjorder et al., 2019). This study also emphasizes the need to approach the diagnosis of genetic diseases in the context of the entire genome rather than key genes, as is common practice for most medical classes (Mahmoud et al., 2023).

## Data Availability

The datasets for this article are not publicly available due to concerns regarding participant/patient anonymity. Requests to access the datasets should be directed to the corresponding author.
