# Supplementary material for: Case Report: Decrypting an interchromosomal insertion associated with Marfan’s syndrome: how optical genome mapping emphasizes the morbid burden of copy-neutral variants
Source: Front Genet. 2023 Sep 21;14:1244983. doi: 10.3389/fgene.2023.1244983 (PMC10551147; doi:10.3389/fgene.2023.1244983)
Supplement: Supplementary file 1 [file DataSheet1.pdf]

**Supplemental material: Decrypting an interchromosomal insertion associated with Marfan's syndrome: how Optical Genome Mapping emphasizes the morbid burden of copy-neutral variants.**

## Tables

**Table S1:** Patient's score for Marfan syndrome according to Ghent criteria (*https://www.ncbi.nlm.nih.gov/books/NBK537339/ )

| Systemic characteristics according to Ghent criteria* | Score |
|---|---|
| Wrist and thumb sign | 3 |
| Pectus excavatum or chest asymmetry | 1 |
| Reduced upper segment/lower segment ratio and increased arm span/height and no severe scoliosis | 1 |
| Scoliosis or thoracolumbar kyphosis | 1 |
| Reduced elbow extension (equal to 170 degrees with full extension) | 1 |
| Mitral valve prolapse | 1 |
| **Total score** | **8** |

**Table S2:** Primers used for Sanger sequencing confirmation of breakpoints junctions (Bjct)

| ID primers | Direction | Sequence (5'->3') | Product length (bp) |
|---|---|---|---|
| Bjct4_15B + 3C _F | Forward | CTTCAACCCAACAGGCTGAATG | 532 |
| Bjct4_15B + 3C _R | Reverse | TAACTTGATTGTGTGCAGTGGCAA | |
| Bjct4_15B + 3C _F | Forward | GGAGGTCTGAACAGGCTACT | 929 |
| Bjct4_15B + 3C_R | Reverse | ACGAAATTTGTTGTTACAGGTGGA | |
| | | | |
| Bjct7_15A + 15C _F | Forward | GGAGAGCTGCTGATGTAACCT | 400 |
| Bjct7_15A + 15C _R | Reverse | TCACGGTTCATTTCACGGGG | |
| Bjct7_15A + 15C _F | Forward | ACGGTGTGGATGGCAAGATT | 717 |
| Bjct7_15A + 15C _R | Reverse | GCTCACGGTTCATTTCACGG | |

**Table S3:** Informative SNPs from the 180K CGH+SNP array platform (G4890A, Agilent Technologies) showing the paternal origin of the 3p12.3 deletion

| SNP ID | Position (hg38) | proband | Father | Mother | Parental origin |
|---|---|---|---|---|---|
| rs2324471 | chr3:76066743 | T | GG | TG | paternal |
| rs7625898 | chr3:76448408 | C | GG | GC | paternal |
| rs11713960 | chr3:77204041 | T | GG | TG | paternal |

**Table S4:** Chromosome fragments participating in the complex chromosome rearrangement (CCR)

| Chromosome | Fragment name | Chromosome band | Fragment lenght | Genome coordinates,hg38 (bps) | Repeats/Genes (orientation) at the left end of the fragment | Repeats/Genes (orientation) at the right end of the fragment | Location in the CCR | Junction partner left (orientation) | Junction partner right (orientation) |
|---|---|---|---|---|---|---|---|---|---|
| 3 | 3A | 3pter-p12.3 | 79,7 Mb | pter-75,798,360 | n.a (telomere) | LINE(L1) -- | der(13) | 13A(+) | n.a (telomere) |
| | 3B | 3p12.3 | 1.7 Mb | 75,798,360-77,574,930 | LTR(ERV)/ -- | --/ROBO2(+) | deleted | -- | -- |
| | 3C | 3p12.3-qter | 120.7 Mb | 77,574,930-qter | --/*ROBO2(+)* | n.a (telomere) | der(3) | 15B(+) | n.a (telomere) |
| 4 | 4A | 4pter-p13 | 44,1 Mb | pter-44,103,646 | n.a (telomere) | LINE(L1)/ -- | der(3) | n.a (telomere) | 13B(+) |
| | 4B | 4p13-qter | 146 Mb | 44,116,764-qter | -- | n.a (telomere) | der(4) | 13D(-) | n.a (telomere) |
| 13 | 13A | 13pter-p12.12 | 23.5 Mb | pter-23,516,377 | n.a (telomere) | --/-- | der(13) | n.a (telomere) | 3A(-) |
| | 13B | 13q12.12 | 939 Kb | 23,532,189-24,478,000 | LTR(ERV1)/ -- | SINE(Alu)-SD/*PARP4*(-) | der(3) | 4A(+) | 13C(-) |
| | 13C | 13q12.2 | 343 Kb | 24,478,000-24,816,560 | SINE(Alu)-SD*/*PARP4(-)* | LINE(L1)/*RNF17*(+) | der(3) | 13B(+) | 15B(+) |
| | 13D | 13q12.2-qter | 89.5 Mb | 24,816,560-qter | LINE(L1)/*RNF17(+)* | n.a (telomere) | der(4) | n.a (telomere) | 4B(+) |
| 15 | 15A | 15pter-q21.1 | 45.7 Mb | pter-45,736,944 | n.a. (telomere) | -- | der(15) | n.a (telomere) | 15C(+) |
| | 15B | 15q21.1 | 2.7 Mb | 45,720,396-48,459,546 | -- | ---/*FBN1(-)* | der(3) | 13C(-) | 3C(+) |
| | 15C | 15q21.1-qter | 53.4 Mb | 48,435,991-qter | --/*FBN1(-)* | n.a (telomere) | der(15) | 15A(+) | n.a (telomere) |

## Figures

**Figure S1:** deletion breakpoints of *ROBO2* estimated by array-CGH (A) and obtained by OGM(C). Genomic coordinates of breakpoints are highlighted within boxes. Panel B shows the structure of *ROBO2* (NM_001395656.1, hg38).

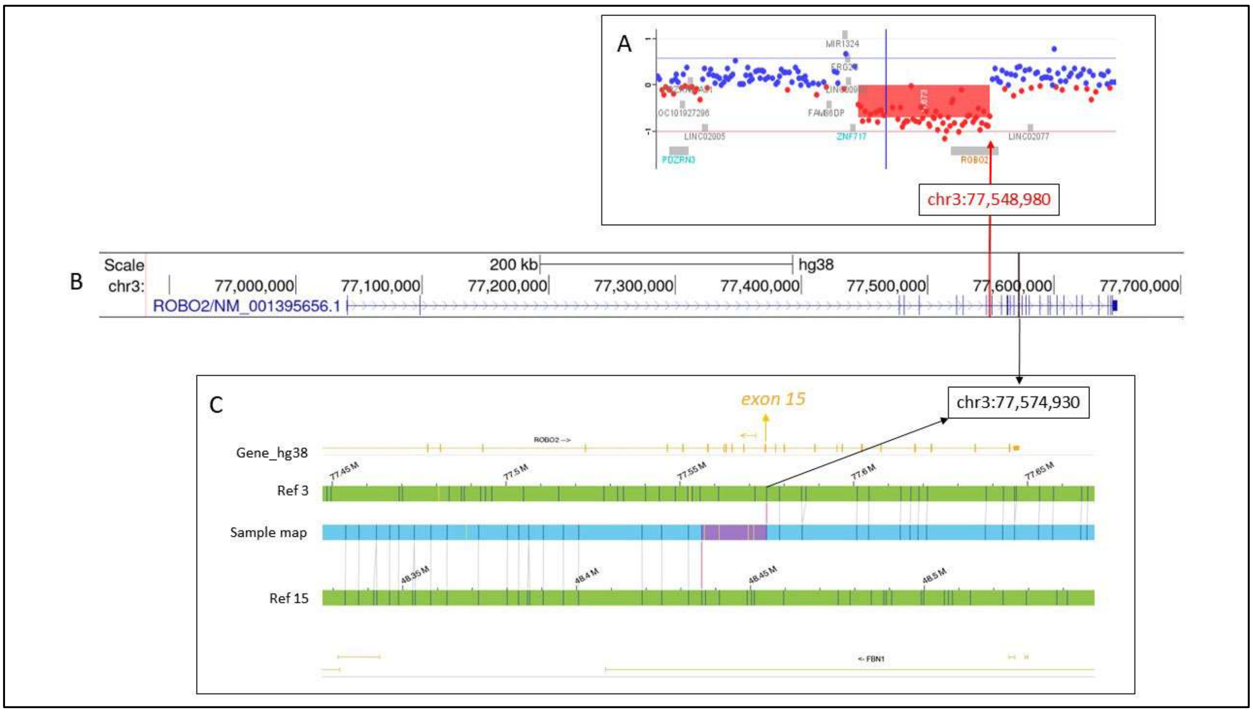

**Figure S2:** FISH on the proband metaphase: probe RP11-552E10 (Genbank Accession: AC012050.7, 15q21.1, spectrum red, Empire Genomics) hybridizes at chr15 and the short arm of the derivative chr3 (arrow point) that is recognizable by the signal of probe CEP3 (chromosome 3 centromere, spectrum Aqua, Empire Genomics).

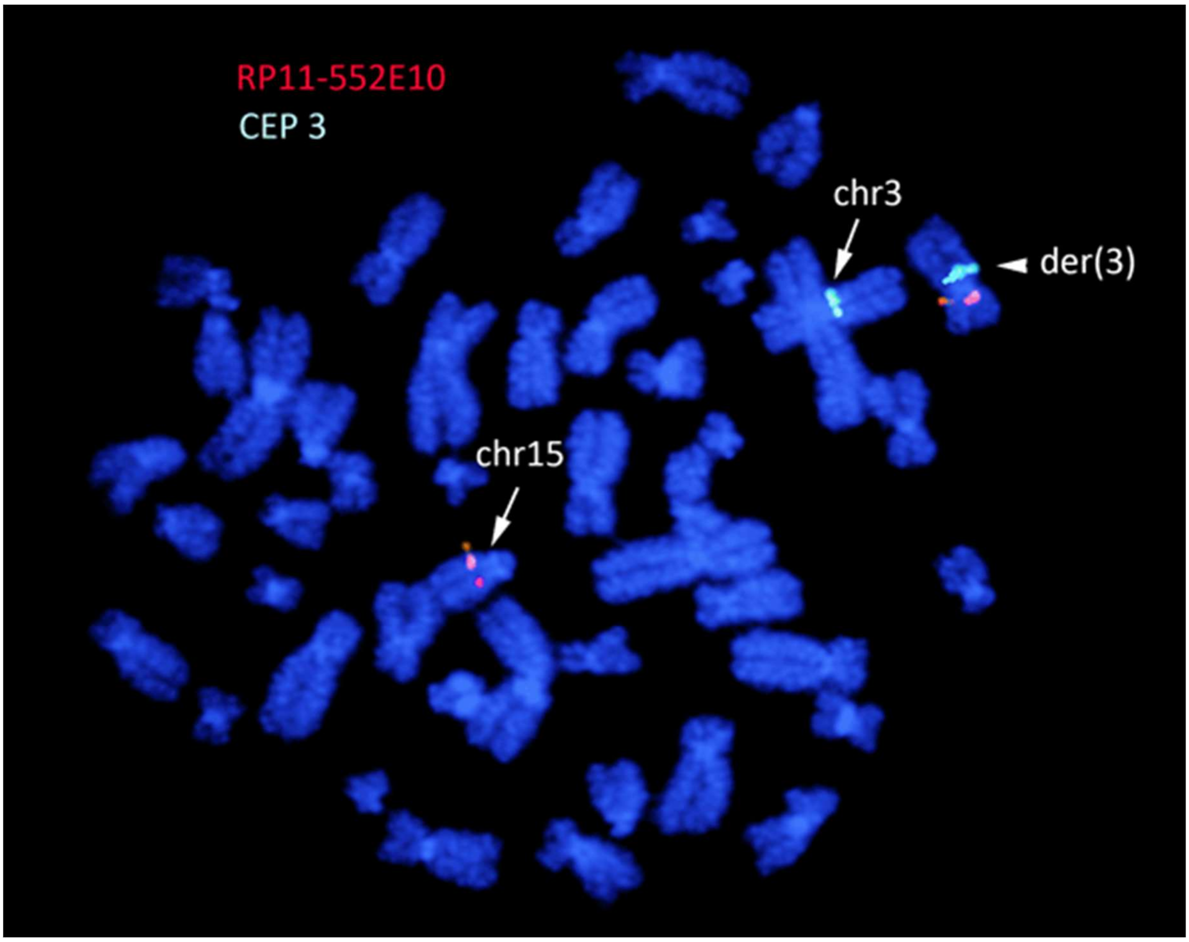

**Figure S3:** Fusion transcript PARP4-RNF17 at sequence breakpoint joining fragments 13B(+) + 13C(-) (see Bjct2 in Table 1)

**Coding sequence of the fusion product of RNF17 (exon 1-15) on fragment 13B (blu) and PARP4 (exon 18-33) on fragment 13C inverted (orange)**

ATGGCGGCAGAGGCTTCGAAGACTGGGCCTTCTAGGTCTTCCTACCAGCGA
ATGGGGAGGAAGAGTCAGCCCTGGGGTGCCGCTGAAATCCAGTGCACCAGG
TGTGGAAGGAGGGTATCCAGATCATCCGGTCACCATTGTGAACTTCAATGT
GGACATGCTTTTTGTGAACTATGCTTGTTAATGACTGAAGAATGCACCACA
ATTATATGCCCTGATTGTGAGGTTGCTACAGCTGTAAATACTAGACAACGC
TACTACCCAATGGCTGGATATATTAAGGAAGACTCCATAATGGAAAAACTG
CAGCCTAAGACGATAAAGAATTGTTCTCAGGACTTTAAGAAGACTGCTGAT
CAGCTAACTACTGGTTTAGAACGTTCAGCCTCCACAGACAAGACTCTTTTG
AACTCATCAGCTGTAATGTTGGACACTAATACTGCAGAAGAAATTGATGAA
GCATTGAATACAGCACACCATAGTTTCGAACAGTTAAGCATTGCTGGAAAA
GCACTTGAACACATGCAGAAGCAAACGATAGAGGAAAGAGAAAGAGTTATA
GAAGTTGTGGAGAAACAGTTTGACCAACTTTTGGCTTTTTTTGATTCCAGG
AAAAAGAACCTGTGTGAAGAATTTGCAAGAACTACTGATGATTATCTATCA
AATTTAATAAAGGCTAAAAGCTACATTGAAGAGAAAAAAAATAATTTGAAT
GCAGCTATGAACATAGCAAGAGCATTACAATTATCGCCTTCTCTAAGAACA
TACTGTGACCTGAATCAGATTATCCGGACTTTGCAGTTAACTTCAGATAGT
GAATTAGCACAAGTTAGTTCTCCACAACTAAGGAACCCTCCCAGGTTGAGT
GTGAATTGCAGTGAGATCATCTGTATGTTCAACAATATGGGAAAGATTGAA
TTTAGGGACTCAACAAAATGTTATCCCCAAGAAAATGAAATTAGACAGAAT
GTTCAAAAGAAATATAATAACAAAAAGGAACTTTCTTGTTACGATACATAC
CCACCGCTAGAAAAGAAAAAGGTTGACATGTCTGTCCTAACCAGTGAAGCA
CCACCACCTCCTTTGCAACCTGAGACAAATGATGTACATTTAGAAGCAAAA
AACTTCCAGCCACAGAAAGACGTTGCAACAGCATCCCCTAAAACCATTGCT
GTGTTACCTCAGATGGGATCTAGCCCTGATGTGATAATTGAAGAAATTATT
GAAGACAACGTGGAAAGTTCTGCAGAGCTAGTTTTTGTAAGCCATGTAATA
GATCCTTGCCATTTCTACATTCGGAAGTATTCACAAATAAAAGACGCCAAA
GTACTGGAGAAGAAGGTGAATGAATTTTGCAATAGGAGTTCACACCTTGAT
CCTTCAGACATTTTGGAACTAGGTGCAAGAATATTTGTCAGCAGTATTAAA
AATGGAATGTGGTGTCGAGGAACTATCACAGAATTAATTCCAATAGAGGGT
AGAAATACCAGAAAACCTTGTAGTCCAACCAGATTATTTGTCCATGAAGTT
GCACTAATACAAATATTCATGGTAGATTTTGGAAATTCTGAAGTCCTGATT
GTCACTGGAGTTGTTGATACCCATGTGAGACCAGAACACTCTGCTAAGCAA
CATATTGCACTAAATGATTTATGTCTGGTTCTAAGGAAATCTGAACCATAT
ACTGAAGGGCTGCTAAAAGACATCCAGCCATTAGCACAACCATGCTCATTG
AAAGACATTGTTCCACAGAATTCAAATGAAGGCTGGGAAGAGGAAGCTAAA
GTGGAATTTTTGAAAATGGTAAATAACAAGGCTGTTTCAATGAAAGTTTTT
AGAGAAGAAGATGGTGTGCTTATTGTAGATCTGCAAAAACCACCACCGAAT
AAAATAAGCAGTGATATGCCTGTGTCTCTTAGAGATGCGCTAGTTTTTATG
GAACTAGCAAAGTTTAAGTCACAATCACTAAGAAGTCACTTTGAAAAAAAT
ACTACTTTACACTATCATCCACCTATTTTGCCTAAAGAAATGACAGATGTT
TCAGTAACGGTTTGTCATATAAATAGTCCTGGAGATTTCTATCTTCAGTTG
GATACAGTAGAGAAGATTTGTATAAAAGAAATAGGAACAAAGCAAAGCTTC
TCTTTGACTATGTCTATTGAGATGCCGTATGTGATTGAATTCATTTTCAGT
GATACACATGAACTGAAACAAAAGCGCACAGACTGCAAAGCTGTCATTAGC
ACCATGGAAGGCAGCTCCTTAGACAGCAGTGGATTTTCTCTCCACATCGGT
TTGTCTGCTGCCTATCTCCCAAGAATGTGGGTTGAAAAACATCCAGAAAAA
GAAAGCGAGGCTTGCATGCTTGTCTTTCAACCCGATCTCGATGTCGACCTC
CCTGACCTAGCCAGTGAGAGCGAAGTGATTATTTGTCTTGACTGCTCCAGT
TCCATGGAGGGTGTGACATTCTTGCAAGCCAAGCAAATCGCCTTGCATGCG
CTGTCCTTGGTGGGTGAGAAGCAGAAAGTAAATATTATCCAGTTCGGCACA
GGTTACAAGGAGCTATTTTCGTATCCTAAGCATATCACAAGCAATACCATG
GCAGCAGAGTTCATCATGTCTGCCACACCTACCATGGGGAACACAGACTTC
TGGAAAACACTCCGATATCTTAGCTTATTGTACCCTGCTCGAGGGTCACGG
AACATCCTCCTGGTGTCTGATGGGCACCTCCAGGATGAGAGCCTGACATTA
CAGCTCGTGAAGAGGAGCCGCCCGCACACCAGGTTATTCGCCTGCGGTATC
GGTTCTACAGCAAATCGTCACGTCTTAAGGATTTTGTCCCAGTGTGGTGCC
GGAGTATTTGAATATTTTAATGCAAAATCCAAGCATAGTTGGAGAAAACAG
ATAGAAGACCAAATGACCAGGCTATGTTCTCCGAGTTGCCACTCTGTCTCC
GTCAAATGGCAGCAACTCAATCCAGATGTGCCCGAGGCCCTGCAGGCCCCA
GCCCAGGTGCCGTCCTTGTTTCTCAATGATCGACTCCTTGTCTATGGATTC
ATTCCTCACTGCACACAGGCAACTCTGTGTGCACTAATTCAAGAGAAAGAA
TTTCGTACAATGGTGTCGACTACTGAGCTTCAGAAGACAACTGGAACTATG
ATCCACAAGCTGGCAGCCCGAGCTCTAATCAGAGATTATGAAGATGGCATT
CTTCACGAAAATGAAACCAGTCATGAGATGAAAAAACAAACCTTGAAATCT
CTGATTATTAAACTCAGTAAAGAAAACTCTCTCATAACACAATTTACAAGC
TTTGTGGCAGTTGAGAAAAGGGATGAGAATGAGTCGCCTTTTCCTGATATT
CCAAAAGTTTCTGAACTTATTGCCAAAGAAGATGTAGACTTCCTGCCCTAC
ATGAGCTGGCAGGGGGAGCCCCAAGAAGCCGTCAGGAACCAGTCTCTTTTA
GCATCCTCTGAGTGGCCAGAATTACGTTTATCCAAACGAAAACATAGGAAA
ATTCCATTTTCCAAAAGAAAAATGGAATTATCTCAGCCAGAAGTTTCTGAA
GATTTTGAAGAGGATGGCTTAGGTGTACTACCAGCTTTCACATCAAATTTG
GAACGTGGAGGTGTGGAAAAGCTATTGGATTTAAGTTGGACAGAGTCATGT
AAACCAACAGCAACTGAACCACTATTTAAGAAAGTCAGTCCATGGGAAACA
TCTACTTCTAGCTTTTTTCCTATTTTGGCTCCGGCCGTTGGTTCCTATCTT
CCCCCGACTGCCCGCGCTCACAGTCCTGCTTCCTTGTCTTTTGCCTCATAT
CGTCAGGTAGCTAGTTTCGGTTCAGCTGCTCCTCCCAGACAGTTTGATGCA
TCTCAATTCAGCCAAGGCCCTGTGCCTGGCACTTGTGCTGACTGGATCCCA
CAGTCGGCGTCTTGTCCCACAGGACCTCCCCAGAACCCACCTTCTTCACCC
TATTGTGGCATTGTTTTTTCAGGGAGCTCATTAAGCTCTGCACAGTCTGCT
CCACTGCAACATCCTGGAGGCTTTACTACCAGGCCTTCTGCTGGCACCTTC
CCTGAGCTGGATTCTCCCCAGCTTCATTTCTCTCTTCCTACAGACCCTGAT
CCCATCAGAGGTTTTGGGTCTTATCATCCCTCTGCTTCCTCTCCTTTTCAT
TTTCAACCTTCCGCAGCCTCTTTGACTGCCAACCTTAGGCTGCCAATGGCC
TCTGCTTTACCTGAGGCTCTTTGCAGTCAGTCCCGGACTACCCCAGTAGAT
CTCTGTCTTCTAGAAGAATCAGTAGGCAGTCTCGAAGGAAGTCGATGTCCT
GTCTTTGCTTTTCAAAGTTCTGACACAGAAAGTGATGAGCTATCAGAAGTA
CTTCAAGACAGCTGCTTTTTACAAATAAAATGTGATACAAAAGATGACAGT
ATCCTGTGCTTTCTGGAAGTAAAAGAAGAGGATGAAATAGTGTGCATACAA
CACTGGCAGGATGCTGTGCCTTGGACAGAACTCCTCAGTCTACAGACAGAG
GATGGCTTCTGGAAACTTACACCAGAACTGGGACTTATATTAAATCTTAAT
ACAAATGGTTTGCACAGCTTTCTTAAACAAAAAGGCATTCAATCTCTAGGT
GTAAAAGGAAGAGAATGTCTCCTGGACCTAATTGCCACAATGCTGGTACTA
CAGTTTATTCGCACCAGGTTGGAAAAAGAGGGAATAGTGTTCAAATCACTG
ATGAAAATGGATGACGCTTCTATTTCCAGGAATATTCCCTGGGCTTTTGAG
GCAATAAAGCAAGCAAGTGAATGGGTAAGAAGAACTGAAGGACAGTACCCA
TCTATCTGCCCACGGCTTGAACTGGGGAACGACTGGGACTCTGCCACCAAG
CAGTTGCTGGGACTCCAGCCCATAAGCACTGTGTCCCCTCTTCATAGAGTC
CTCCATTACAGTCAAGGCTAA

**Potential open reading frames (ORFs) of the fusion product assessed by the ExPASy's Translation Tool are red-highlighted; horizontal dashes represent a (premature) stop codon (http://web.expasy.org/translate/)**

5'3' Frame 1
MAAEASKTGPSRSSYQRMGRKSQPWGAAEIQCTRCGRRVSRSSGHHCELQC
GHAFCELCLLMTEECTTIICPDCEVATAVNTRQRYYPMAGYIKEDSIMEKL
QPKTIKNCSQDFKKTADQLTTGLERSASTDKTLLNSSAVMLDTNTAEEIDE
ALNTAHHSFEQLSIAGKALEHMQKQTIEERERVIEVVEKQFDQLLAFFDSR
KKNLCEEFARTTDDYLSNLIKAKSYIEEKKNNLNAAMNIARALQLSPSLRT
YCDLNQIIRTLQLTSDSELAQVSSPQLRNPPRLSVNCSEIICMFNNMGKIE
FRDSTKCYPQENEIRQNVQKKYNNKKELSCYDTYPPLEKKKVDMSVLTSEA
PPPPLQPETNDVHLEAKNFQPQKDVATASPKTIAVLPQMGSSPDVIIEEII
EDNVESSAELVFVSHVIDPCHFYIRKYSQIKDAKVLEKKVNEFCNRSSHLD
PSDILELGARIFVSSIKNGMWCRGTITELIPIEGRNTRKPCSPTRLFVHEV
ALIQIFMVDFGNSEVLIVTGVVDTHVRPEHSAKQHIALNDLCLVLRKSEPY
TEGLLKDIQPLAQPCSLKDIVPQNSNEGWEEEAKVEFLKMVNNKAVSMKVF
REEDGVLIVDLQKPPPNKISSDMPVSLRDALVFMELAKFKSQSLRSHFEKN
TTLHYHPPILPKEMTDVSVTVCHINSPGDFYLQLDTVEKICIKEIGTKQSF
SLTMSIEMPYVIEFIFSDTHELKQKRTDCKAVISTMEGSSLDSSGFSLHIG
LSAAYLPRMWVEKHPEKESEACMLVFQPDLDVDLPDLASESEVIICLDCSS
SMEGVTFLQAKQIALHALSLVGEKQKVNIIQFGTGYKELFSYPKHITSNTM
AAEFIMSATPTMGNTDFWKTLRYLSLLYPARGSRNILLVSDGHLQDESLTL
QLVKRSRPHTRLFACGIGSTANRHVLRILSQCGAGVFEYFNAKSKHSWRKQ
IEDQMTRLCSPSCHSVSVKWQQLNPDVPEALQAPAQVPSLFLNDRLLVYGF
IPHCTQATLCALIQEKEFRTMVSTTELQKTTGTMIHKLAARALIRDYEDGI
LHENETSHEMKKQTLKSLIIKLSKENSLITQFTSFVAVEKRDENESPFPDI
PKVSELIAKEDVDFLPYMSWQGEPQEAVRNQSLLASSEWPELRLSKRKHRK
IPFSKRKMELSQPEVSEDFEEDGLGVLPAFTSNLERGGVEKLLDLSWTESC
KPTATEPLFKKVSPWETSTSSFFPILAPAVGSYLPPTARAHSPASLSFASY
RQVASFGSAAPPRQFDASQFSQGPVPGTCADWIPQSASCPTGPPQNPPSSP
YCGIVFSGSSLSSAQSAPLQHPGGFTTRPSAGTFPELDSPQLHFSLPTDPD
PIRGFGSYHPSASSPFHFQPSAASLTANLRLPMASALPEALCSQSRTTPVD
LCLLEESVGSLEGSRCPVFAFQSSDTESDELSEVLQDSCFLQIKCDTKDDS
ILCFLEVKEEDEIVCIQHWQDAVPWTELLSLQTEDGFWKLTPELGLILNLN
TNGLHSFLKQKGIQSLGVKGRECLLDLIATMLVLQFIRTRLEKEGIVFKSL
MKMDDASISRNIPWAFEAIKQASEWVRRTEGQYPSICPRLELGNDWDSATK
QLLGLQPISTVSPLHRVLHYSQG-

5'3' Frame 2
WRQRLRRLGLLGLPTSEWGGRVSPGVPLKSSAPGVEGGYPDHPVTIVNFNV
DMLFVNYAC--LKNAPQLYALIVRLLQL-ILDNATTQWLDILRKTP-WKNC
SLRR-RIVLRTLRRLLIS-LLV-NVQPPQTRLF-THQL-CWTLILQKKLMK
H-IQHTIVSNS-ALLEKHLNTCRSKR-RKEKEL-KLWRNSLTNFWLFLIPG
KRTCVKNLQELLMIIYQI--RLKATLKRKKII-MQL-T-QEHYNYRLL-EH
TVT-IRLSGLCS-LQIVN-HKLVLHN-GTLPG-V-IAVRSSVCSTIWERLN
LGTQQNVIPKKMKLDRMFKRNIITKRNFLVTIHTHR-KRKRLTCLS-PVKH
HHLLCNLRQMMYI-KQKTSSHRKTLQQHPLKPLLCYLRWDLALM--LKKLL

KTTWKVLQS-FL-AM--ILAISTFGSIHK-KTPKYWRRR-MNFAIGVHTLI
LQTFWN-VQEYLSAVLKMECGVEELSQN-FQ-RVEIPENLVVQPDYLSMKL
H-YKYSW-ILEILKS-LSLELLIPM-DQNTLLSNILH-MIYVWF-GNLNHI
LKGC-KTSSH-HNHAH-KTLFHRIQMKAGKRKLKWNF-KW-ITRLFQ-KFL
EKKMVCLL-ICKNHHRIK-AVICLCLLEMR-FLWN-QSLSHNH-EVTLKKI
LLYTIIHLFCLKK-QMFQ-RFVI-IVLEISIFSWIQ-RRFV-KK-EQSKAS
L-LCLLRCRM-LNSFSVIHMN-NKSAQTAKLSLAPWKAAP-TAVDFLSTSV
CLLPISQECGLKNIQKKKARLACLSFNPISMSTSLT-PVRAK-LFVLTAPV
PWRV-HSCKPSKSPCMRCPWWVRSRK-ILSSSAQVTRSYFRILSISQAIPW
QQSSSCLPHLPWGTQTSGKHSDILAYCTLLEGHGTSSWCLMGTSRMRA-HY
SS-RGAARTPGYSPAVSVLQQIVTS-GFCPSVVPEYLNILMQNPSIVGENR
-KTK-PGYVLRVATLSPSNGSNSIQMCPRPCRPQPRCRPCFSMIDSLSMDS
FLTAHRQLCVH-FKRKNFVQWCRLLSFRRQLEL-STSWQPEL-SEIMKMAF
FTKMKPVMR-KNKP-NL-LLNSVKKTLS-HNLQALWQLRKGMRMSRLFLIF
QKFLNLLPKKM-TSCPT-AGRGSPKKPSGTSLF-HPLSGQNYVYPNENIGK
FHFPKEKWNYLSQKFLKILKRMA-VYYQLSHQIWNVEVWKSYWI-VGQSHV
NQQQLNHYLRKSVHGKHLLLAFFLFWLRPLVPIFPRLPALTVLLPCLLPHI
VR-LVSVQLLLPDSLMHLNSAKALCLALVLTGSHSRRLVPQDLPRTHLLHP
IVALFFQGAH-ALHSLLHCNILEALLPGLLLAPSLSWILPSFISLFLQTLI
PSEVLGLIIPLLPLLFIFNLPQPL-LPTLGCQWPLLYLRLFAVSPGLPQ-I
SVF-KNQ-AVSKEVDVLSLLFKVLTQKVMSYQKYFKTAAFYK-NVIQKMTV
SCAFWK-KKRMK-CAYNTGRMLCLGQNSSVYRQRMASGNLHQNWDLY-ILI
QMVCTAFLNKKAFNL-V-KEENVSWT-LPQCWYYSLFAPGWKKRE-CSNH-
-KWMTLLFPGIFPGLLRQ-SKQVNG-EELKDSTHLSAHGLNWGTTGTLPPS
SCWDSSP-ALCPLFIESSITVKA

5'3' Frame 3
GGRGFEDWAF-VFLPANGEEESALGCR-NPVHQVWKEGIQIIRSPL-TSMW
TCFL-TMLVND-RMHHNYMP-L-GCYSCKY-TTLLPNGWIY-GRLHNGKTA
A-DDKELFSGL-EDC-SANYWFRTFSLHRQDSFELISCNVGH-YCRRN--S
IEYSTP-FRTVKHCWKST-THAEANDRGKRKSYRSCGETV-PTFGFF-FQE
KEPV-RICKNY--LSIKFNKG-KLH-REKK-FECSYEHSKSITIIAFSKNI
L-PESDYPDFAVNFR--ISTS-FSTTKEPSQVECELQ-DHLYVQQYGKD-I
-GLNKMLSPRK-N-TECSKEI--QKGTFLLRYIPTARKEKG-HVCPNQ-ST
TTSFAT-DK-CTFRSKKLPATERRCNSIP-NHCCVTSDGI-P-CDN-RNY-
RQRGKFCRASFCKPCNRSLPFLHSEVFTNKRRQSTGEEGE-ILQ-EFTP-S
FRHFGTRCKNICQQY-KWNVVSRNYHRINSNRG-KYQKTL-SNQIICP-SC
TNTNIHGRFWKF-SPDCHWSC-YPCETRTLC-ATYCTK-FMSGSKEI-TIY
-RAAKRHPAISTTMLIERHCSTEFK-RLGRGS-SGIFENGK-QGCFNESF-
RRRWCAYCRSAKTTTE-NKQ-YACVS-RCASFYGTSKV-VTITKKSL-KKY
YFTLSSTYFA-RNDRCFSNGLSYK-SWRFLSSVGYSREDLYKRNRNKAKLL
FDYVY-DAVCD-IHFQ-YT-TETKAHRLQSCH-HHGRQLLRQQWIFSPHRF
VCCLSPKNVG-KTSRKRKRGLHACLSTRSRCRPP-PSQ-ERSDYLS-LLQF
HGGCDILASQANRLACAVLGG-EAESKYYPVRHRLQGAIFVS-AYHKQYHG
SRVHHVCHTYHGEHRLLENTPIS-LIVPCSRVTEHPPGV-WAPPG-EPDIT
AREEEPPAHQVIRLRYRFYSKSSRLKDFVPVWCRSI-IF-CKIQA-LEKTD
RRPNDQAMFSELPLCLRQMAATQSRCARGPAGPSPGAVLVSQ-STPCLWIH
SSLHTGNSVCTNSRERISYNGVDY-ASEDNWNYDPQAGSPSSNQRL-RWHS
SRK-NQS-DEKTNLEISDY-TQ-RKLSHNTIYKLCGS-EKG-E-VAFS-YS
KSF-TYCQRRCRLPALHELAGGAPRSRQEPVSFSIL-VARITFIQTKT-EN
SIFQKKNGIISARSF-RF-RGWLRCTTSFHIKFGTWRCGKAIGFKLDRVM-
TNSN-TTI-ESQSMGNIYF-LFSYFGSGRWFLSSPDCPRSQSCFLVFCLIS
SGS-FRFSCSSQTV-CISIQPRPCAWHLC-LDPTVGVLSHRTSPEPTFFTL
LWHCFFRELIKLCTVCSTATSWRLYYQAFCWHLP-AGFSPASFLSSYRP-S
HQRFWVLSSLCFLSFSFSTFRSLFDCQP-AANGLCFT-GSLQSVPDYPSRS
LSSRRISRQSRRKSMSCLCFSKF-HRK--AIRSTSRQLLFTNKM-YKR-QY
PVLSGSKRRG-NSVHTTLAGCCALDRTPQSTDRGWLLETYTRTGTYIKS-Y
KWFAQLS-TKRHSISRCKRKRMSPGPNCHNAGTTVYSHQVGKRGNSVQITD
ENG-RFYFQEYSLGF-GNKASK-MGKKN-RTVPIYLPTA-TGERLGLCHQA
VAGTPAHKHCVPSS-SPPLQSRL

**The first frame generates a long uninterrupted ORF resulting in a putative protein fusion by ScanProsite(https://prosite.expasy.org/scanprosite/)**

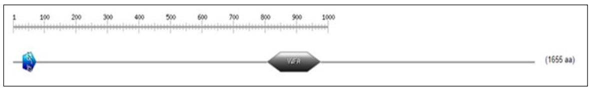

**Figure S4** IGV exploration of the fragments from chromosomes 4, 13, and 15 that compose the derivative chromosome 3. Dashed vertical lines indicate breakpoints of each fragment. Panels A-D show the mate regions of the four breakpoints junction (see Table1 and Figure 2 in the main text) identified by OGM (see Table S3). Discordant reads that map to the left (A, B) or right (C, D) region of the breakpoint have mate pairs that map to the positive (+) or negative (-) strand at a second chromosome region. Genomic coordinates of soft-clipped reads are highlighted within boxes. Breakpoint junction sequences, of which two characterized by microhomology of 3-5 bps (A, C) and one by blunt ends (B), are indicated.

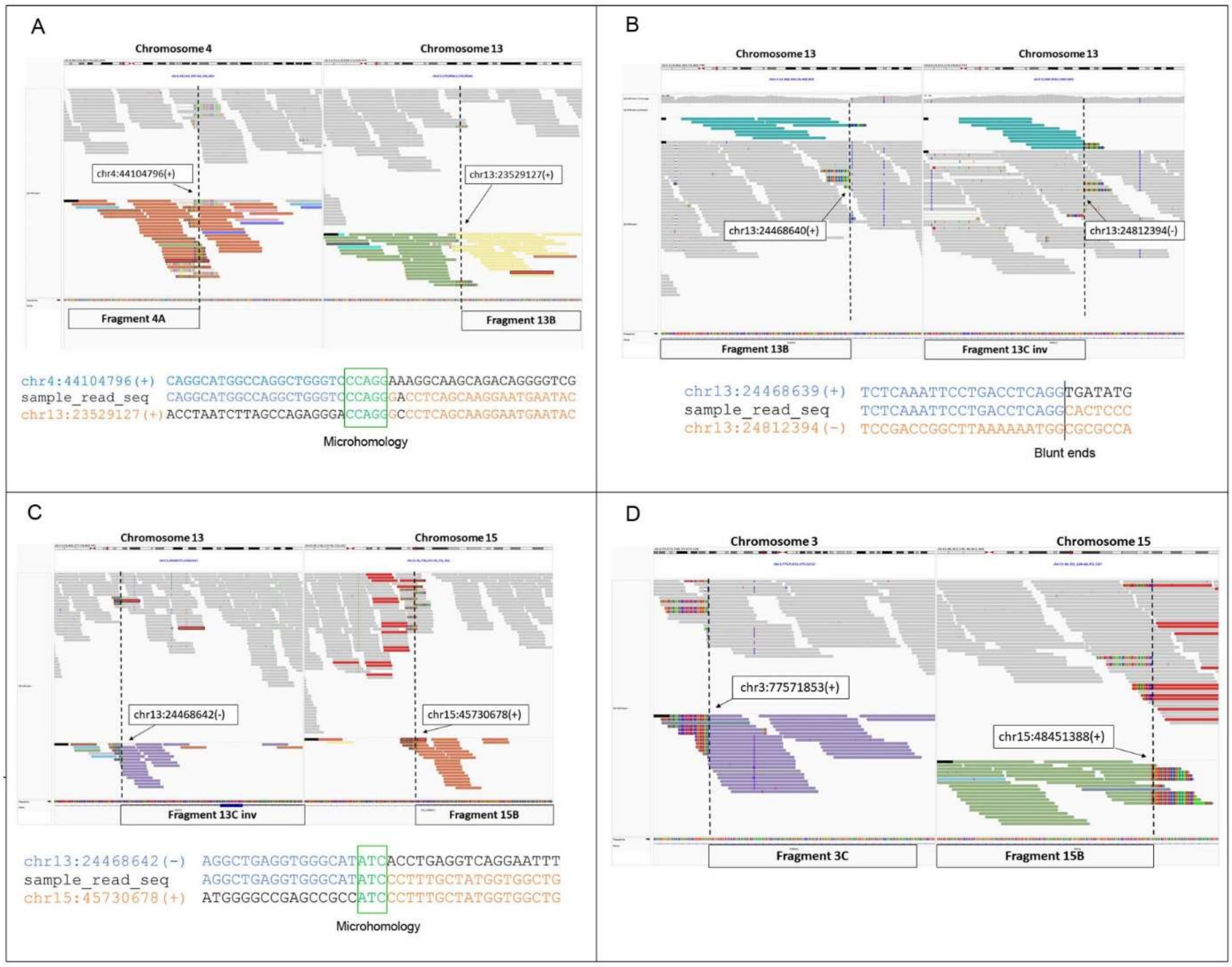

**Figure S5** IGV exploration of the fragments 4B and 13D that compose the derivative chromosome 4. Dashed vertical lines indicate the breakpoints of each fragment as identified by OGM. Soft-clipped reads that map to the right side of the breakpoint at chr4:44104800 (+) have mate pair located to negative (-) strand of chromosome 13 (fragment 13D inv). Blast results of soft-clipped read sequences at chromosome 13 were not informative due to the presence of repeated sequences.

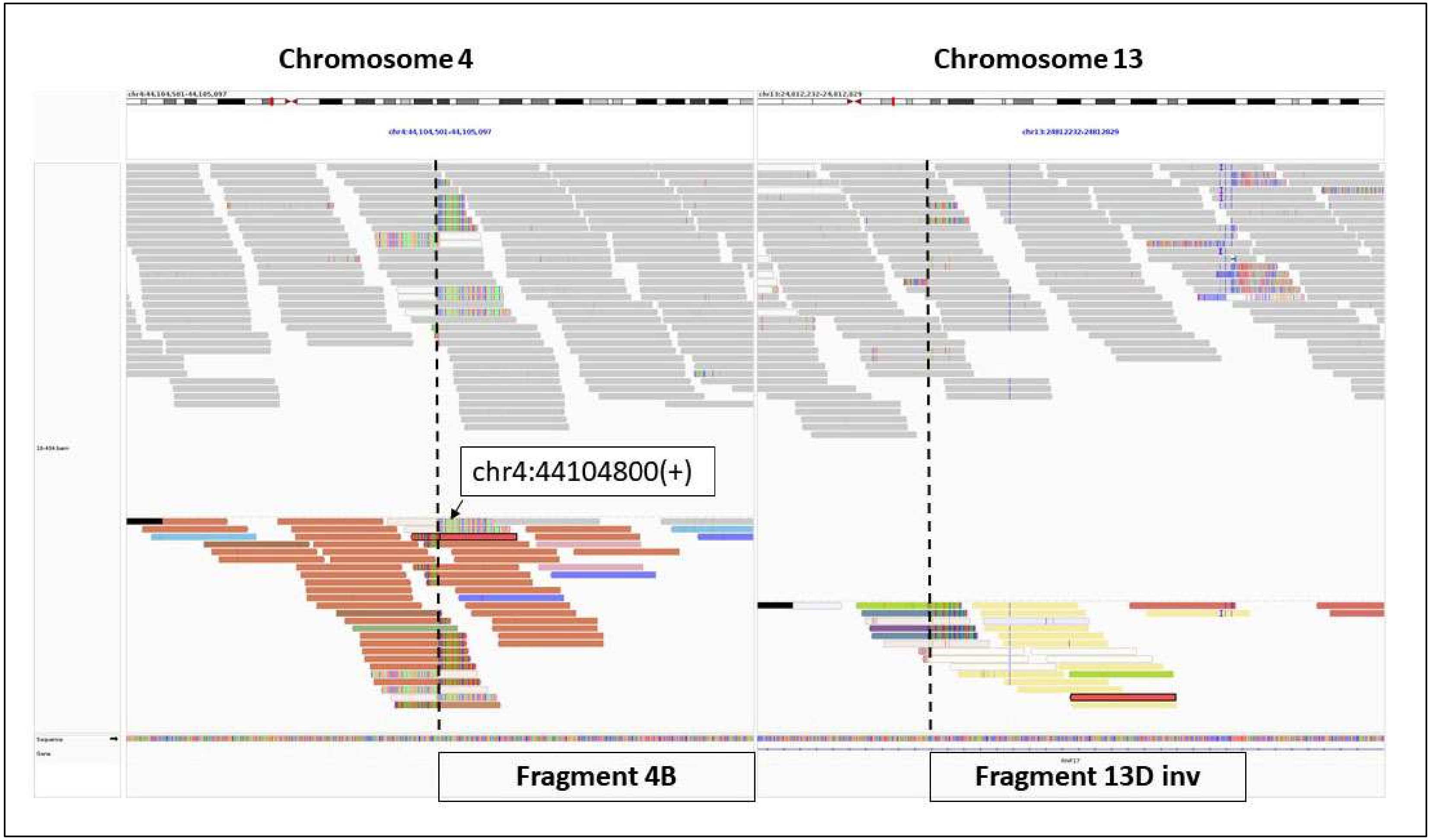

**Figure S6** IGV visualization of fragments 3Ainv and 13A (indicated with rectangles) that compose the derivative chromosome 13. Dashed vertical lines indicate the breakpoints of each fragment as identified by OGM. Soft-clipped reads that map to the left side of the breakpoint at chr13:23529137(+) have mate pair located to negative (-) strand of chromosome 3 (fragment 13Ainv). Blast results of soft-clipped read sequences at chromosome 3 were not informative due to the presence of repeated sequences.

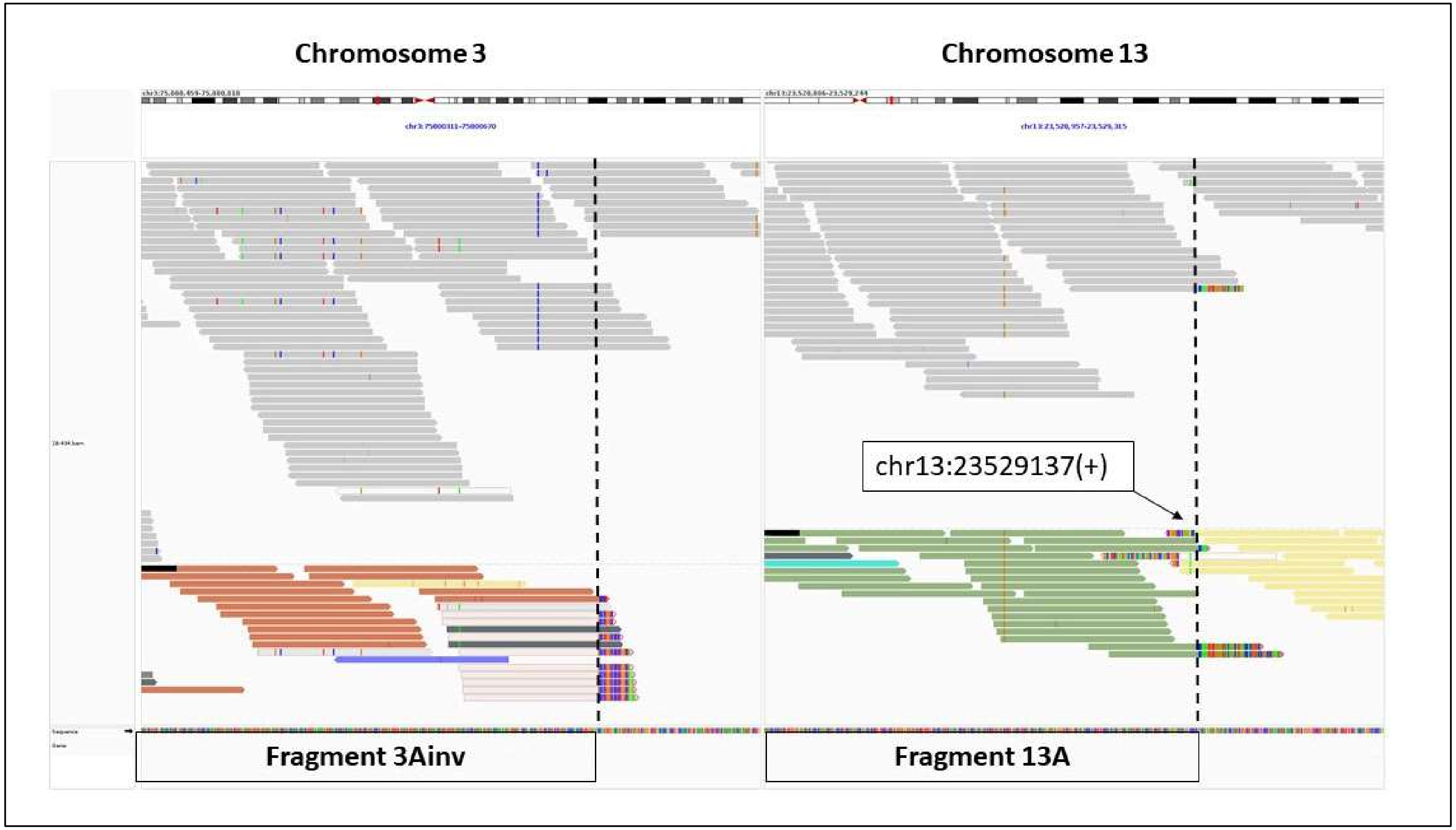

**Figure S7** IGV visualization of chromosome 15, chr15:45,730,501-45,730,820 (left) and chr15:48,451,366-48,451,686 (right) including fragments 15A and 15C. Soft-clipped reads (red) that map to the left side of the breakpoint at chr15:45,730,661 (+) have mate pair to positive (+) strand at chr 15:48,451,391, consistent with breakpoint junction 15A(+) +15C(+) previously identified by OGM and verified by Sanger sequencing as shown in Figure 2F.

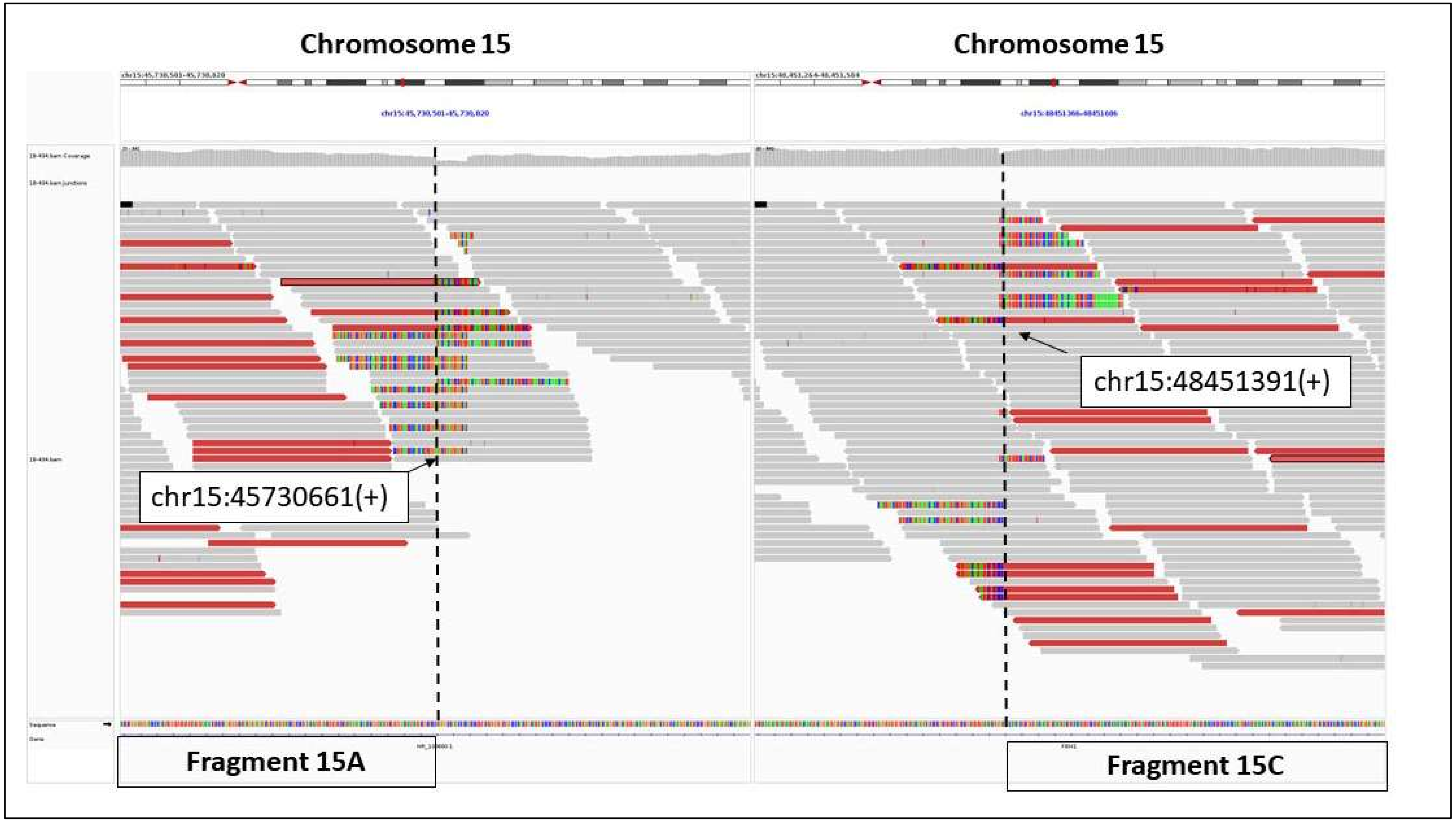

**Figure S8 Structural domains and mutation mapping of ROBO2**. A schematic representation of the domain structure of ROBO2 is shown. Domain information was retrieved from UniProt (https://www.uniprot.org/) and refers to the Q9HCK4 entry. The location of intrinsically disordered regions is also shown. Missense mutations associated with VUR (black) or CAKUT (congenital anomalies of the kidney and urinary tract, blue) were derived from ClinVar (https://www.ncbi.nlm.nih.gov/clinvar/ ) and are mapped onto the domain structure of the protein. The extension of the deletion is reported.

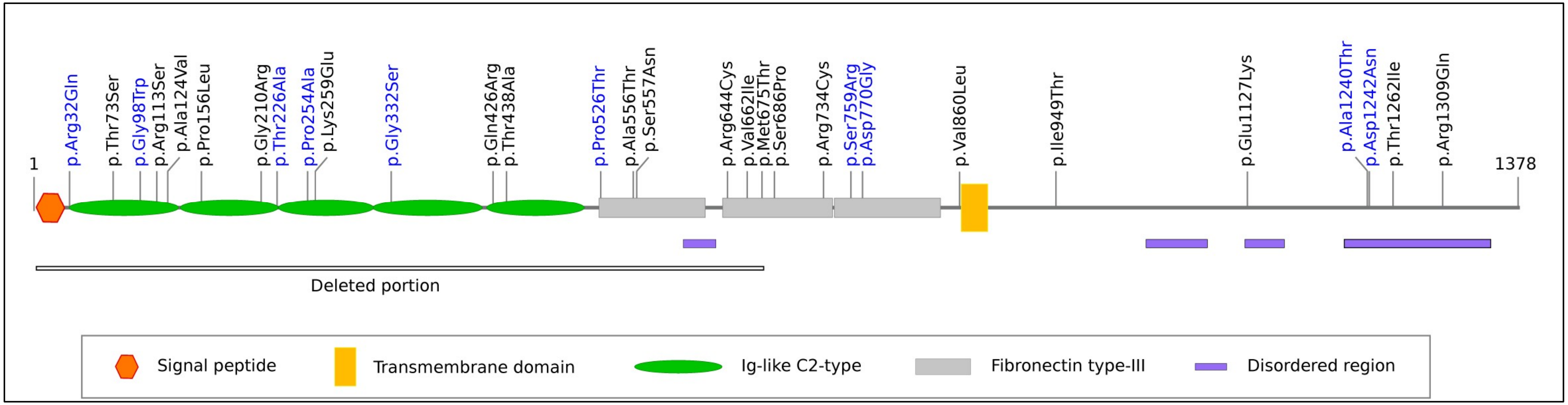

**Figure S9** Schematic illustration of the Topologically Associated Domain (TAD) structure encompassing the fragments (indicated with a horizontal gray and red bars) forming the rearrangement (see Table S3), as created by the 3D Genome Browser (http://promoter.bx.psu.edu/hi-c/). The GM12878 Hi-C maps (Ro et al 2014; left) and H1-ESC (Dixon et al. 2015, right) are shown for each schematic view of TADs. The corresponding region from the UCSC Genome Browser was aligned underneath the heat map.

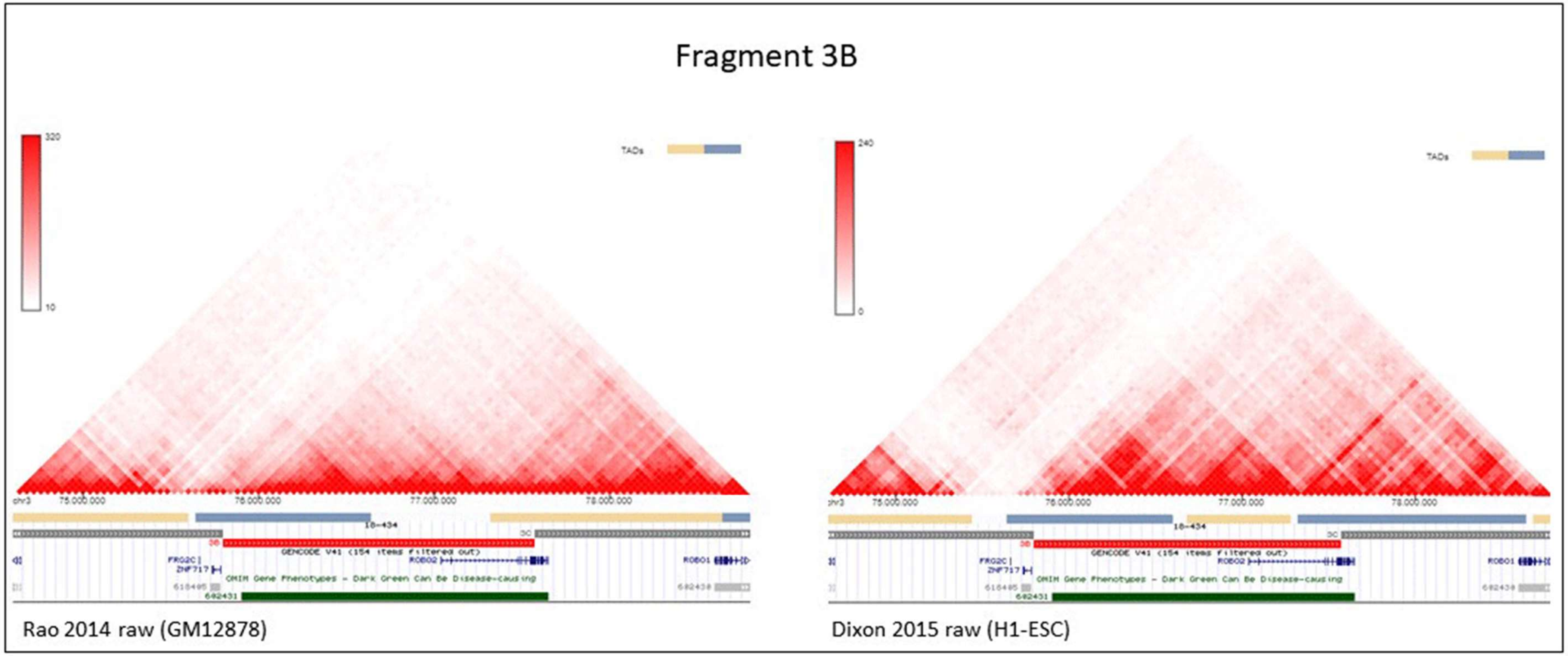

## Fragments 4A-4B

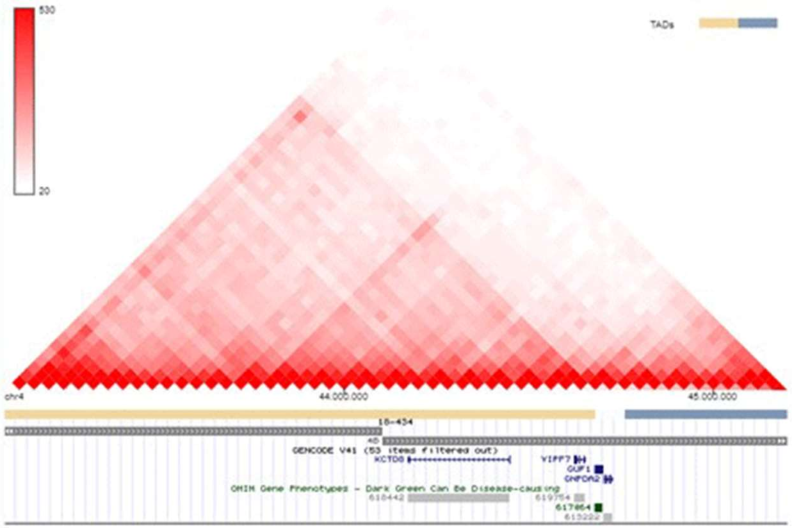


Rao 2014 raw (GM12878)

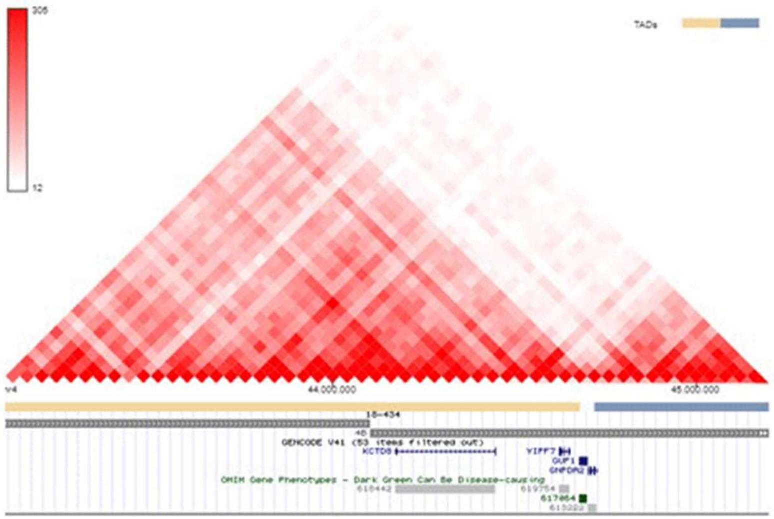


Dixon 2015 raw (H1-ESC)

## Fragments 13B-13C

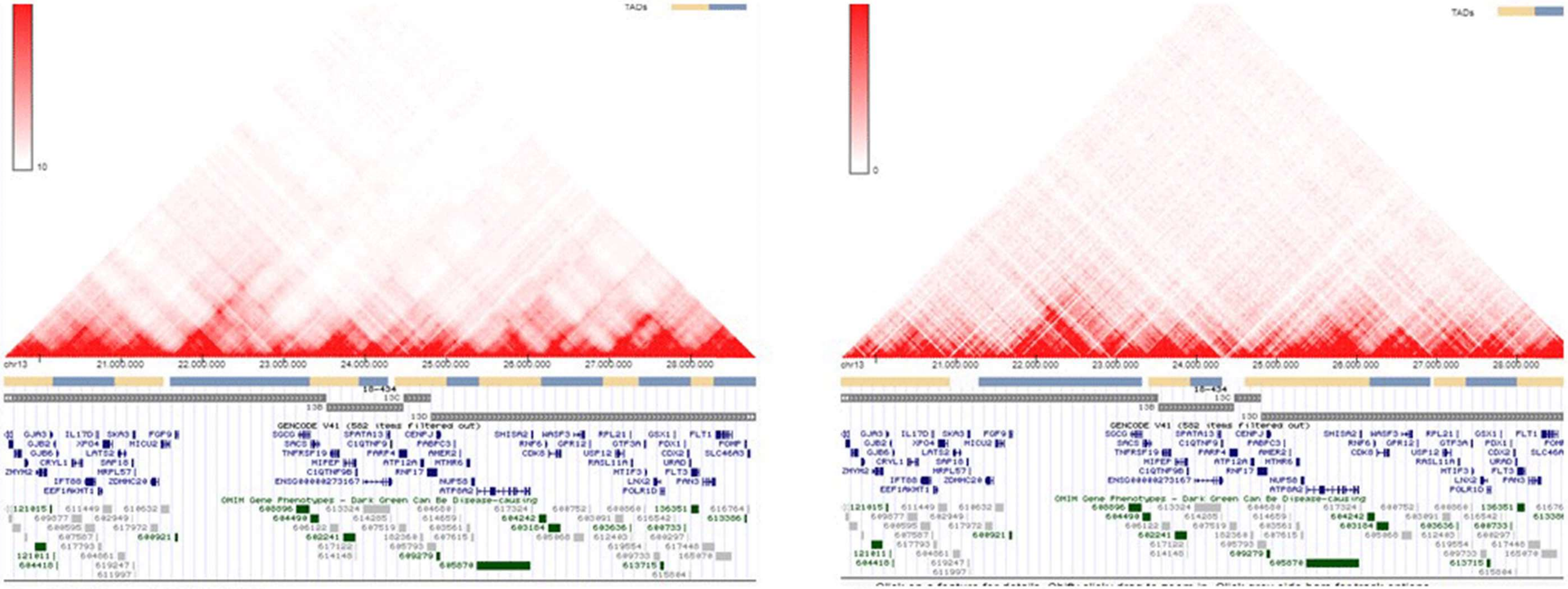


Rao 2014 raw (GM12878)        Dixon 2015 raw (H1-ESC)

## Fragment 15B

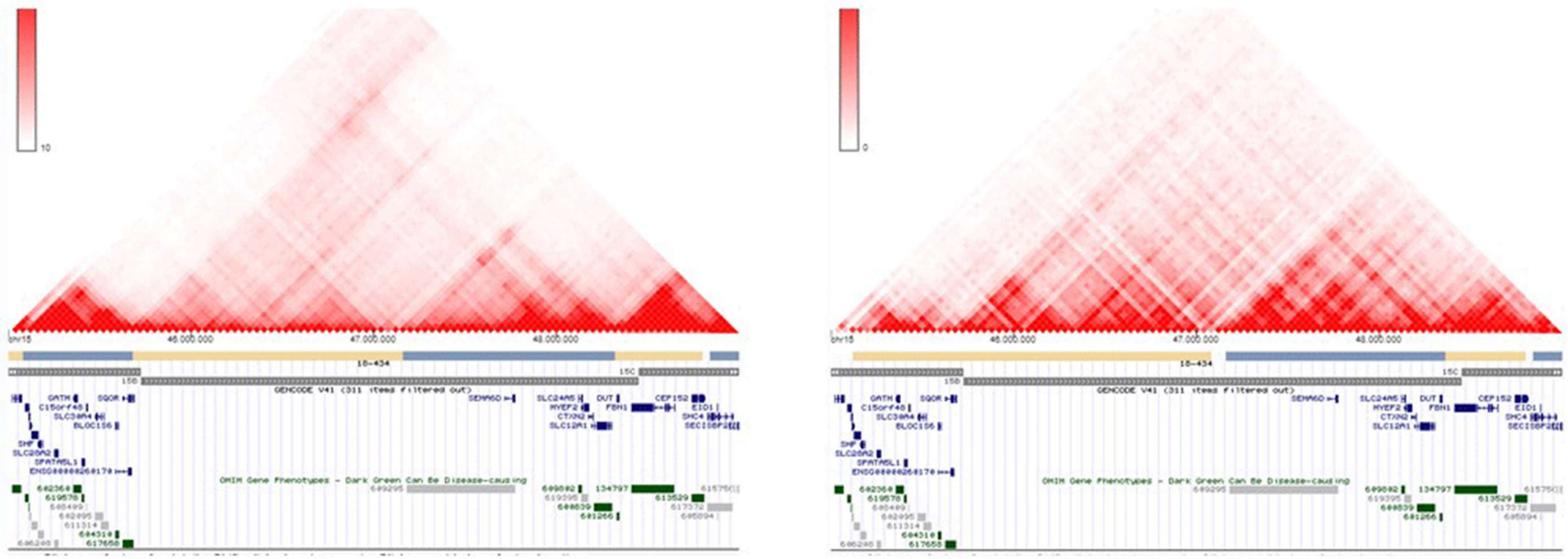


Rao 2014 raw (GM12878)

Dixon 2015 raw (H1-ESC)
